# Challenges in Management of Compromised First Permanent Molar With Pathologic Perforation and Periapical Lesion in a 10-Year-Old Boy: A Case Report

**DOI:** 10.7759/cureus.85990

**Published:** 2025-06-14

**Authors:** Sahili Mungekar, Laresh N Mistry, Ashwin M Jawdekar, Shreyas Neelkanthan, Snehal Markandey, Punam S Patil

**Affiliations:** 1 Department of Pediatric and Preventive Dentistry, Vidarbha Youth Welfare Society (VYWS) Dental College and Hospital, Amravati, IND; 2 Department of Pediatric and Preventive Dentistry, Bharati Vidyapeeth (Deemed to be University) Dental College and Hospital, Navi Mumbai, IND

**Keywords:** case-report, challenges, compromised tooth, first permanent molar, healing of preapical lesion, intracanal medicament, pathologic perforation, periapical lesion, triple antibiotic paste

## Abstract

First permanent molars play a critical role in mastication, development of occlusion, dentoalveolar growth and maintenance of dentofacial and skeletal harmony.

The first permanent molar is the most caries-prone tooth in the permanent dentition due to its early exposure to the oral environment. Additionally, it takes the longest time to develop from its intrauterine formation to eruption in the oral cavity making it particularly susceptible to hypoplastic changes during development. It has been frequently observed that the age of the child, stage of growth and development, incomplete root formation, limited working area in pediatric patients, behavioural changes, and delayed reporting to the dental clinic complicate the management and affect the long-term prognosis of the molar.

Management of compromised first permanent molars (cFPM) in pediatric patients presents several challenges, including behavioural issues, limited cooperation, incomplete root formation, restricted working space, and delayed presentation to dental clinics. These factors, along with difficulties in achieving effective local anesthesia, poor saliva control, and the rapid progression of carious lesions, further complicate treatment and affect the long-term prognosis.

The present paper describes the management of compromised first permanent molar in a 10-year-old boy with carious and pathologic furcation involvement and compromised periodontal prognosis using Triple antibiotic paste, Tricalcium silicate cement, intra-operative and post-operative challenges during its management. At follow-up, the tooth was evaluated clinically for the presence of pain, swelling, and difficulty in mastication. Radiographic assessment was performed over a specified time period to monitor the resolution of the periapical lesion and to confirm signs of bone healing and re-establishment of the lamina dura.

## Introduction

Compromised first permanent molars (cFPM) affect the overall oral health and quality of life in children [1,2]. First permanent molars play a critical role in mastication, development of occlusion, dentoalveolar growth, and maintenance of dentofacial and skeletal harmony [3].

The mean global prevalence of molar incisor hypomineralisation (MIH) is 13% [4]. Dental caries and MIH are considered as the most common aetiological factors that render a first permanent molar to be of compromised prognosis [5]. The first permanent molar (FPM) not only erupts at the age of six years but has also been quoted as being the most caries-prone tooth in the permanent dentition, because of its early exposure to the oral environment [6]. Also, it takes the longest time to develop from its developmental intra-uterine position to erupt in the oral cavity, so it is most susceptible to developmental influences and changes. Management of such compromised first permanent molar (cFPM) is a challenge to the clinician due to a lack of cooperation in children, injection-related anxiety and fear, inadequate action of anesthesia, and rapid spread of carious lesion because newly erupted teeth are more permeable and less mineralized, allowing rapid diffusion of acids [3].

Conservative treatment of compromised permanent multirooted teeth presented for endodontic management poses a challenge to achieve adequate disinfection and a three-dimensional apical seal due to factors such as incomplete root formation, thin radicular dentin, larger and more permeable dentinal tubules, and wider root canals [3]. Endodontic management of permanent teeth may involve procedural complications such as instrument separation, canal zipping, ledge formation, and coronal or radicular perforations. These perforations can be either iatrogenic in origin or pathologic, often arising from chronic inflammatory processes [5]. Perforations can be managed by two approaches, either radically by extraction or by sealing the perforation site with a biocompatible material that can address the associated infection and inflammation to provide a hermetic seal and prevent reinfection. If extraction is considered as the treatment modality, the ideal time to extract first permanent molars is between 8 and 10 years of age, typically when the tooth is at Demirjian stage E [6]. Many other factors may influence the decision-making of treatment in young children, such as age, extent of caries, periapical pathology, condition of dentition, financial implications, co-operation compliance of the patient and parent consent.

Perforation site management is based on two principles: disinfection and inflammation management, and three-dimensional repair of the perforation site with restorative or biocompatible material. Various perforation repair restorative materials in permanent teeth include amalgam, resin-modified glass ionomer cement (RMGIC), composite, and biocompatible materials, including Portland cement, mineral trioxide aggregate (MTA), and Biodentine™ (Septodont, Saint-Maur-des-Fossés Cedex, France) [7]. Medicaments such as calcium hydroxide (CaOH2) or calcium hydroxide with iodoform were used traditionally to disinfect the perforation site, followed by sealing with amalgam or RMGIC. Recently, materials such as MTA, calcium-enriched mixture (CEM), Biodentine™, and other bioactive materials have combined disinfectant and anti-inflammatory properties along with good sealing ability [8].

The concept of Lesion Sterilization and Tissue Repair (LSTR) was given by Hoshino, tested by Sato et al., and popularized by Takushige et al., and was found effective in sterilizing carious lesions, infected dentin, and periapical and periradicular infections [9-11].

This case report aims to describe the endodontic management of a compromised first permanent molar with carious and pathologic perforation, using triple antibiotic paste (TAP) as the intracanal medicament, followed by obturation and perforation repair with Biodentine™, a biocompatible material.

## Case presentation

**Figure 1:**
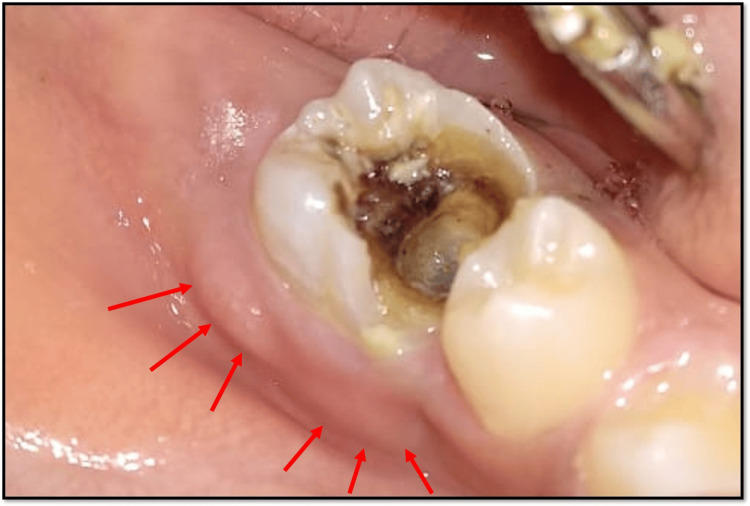
Pre-treatment intraoral clinical view showing deep occlusal caries with respect to 46

**Figure 2:**
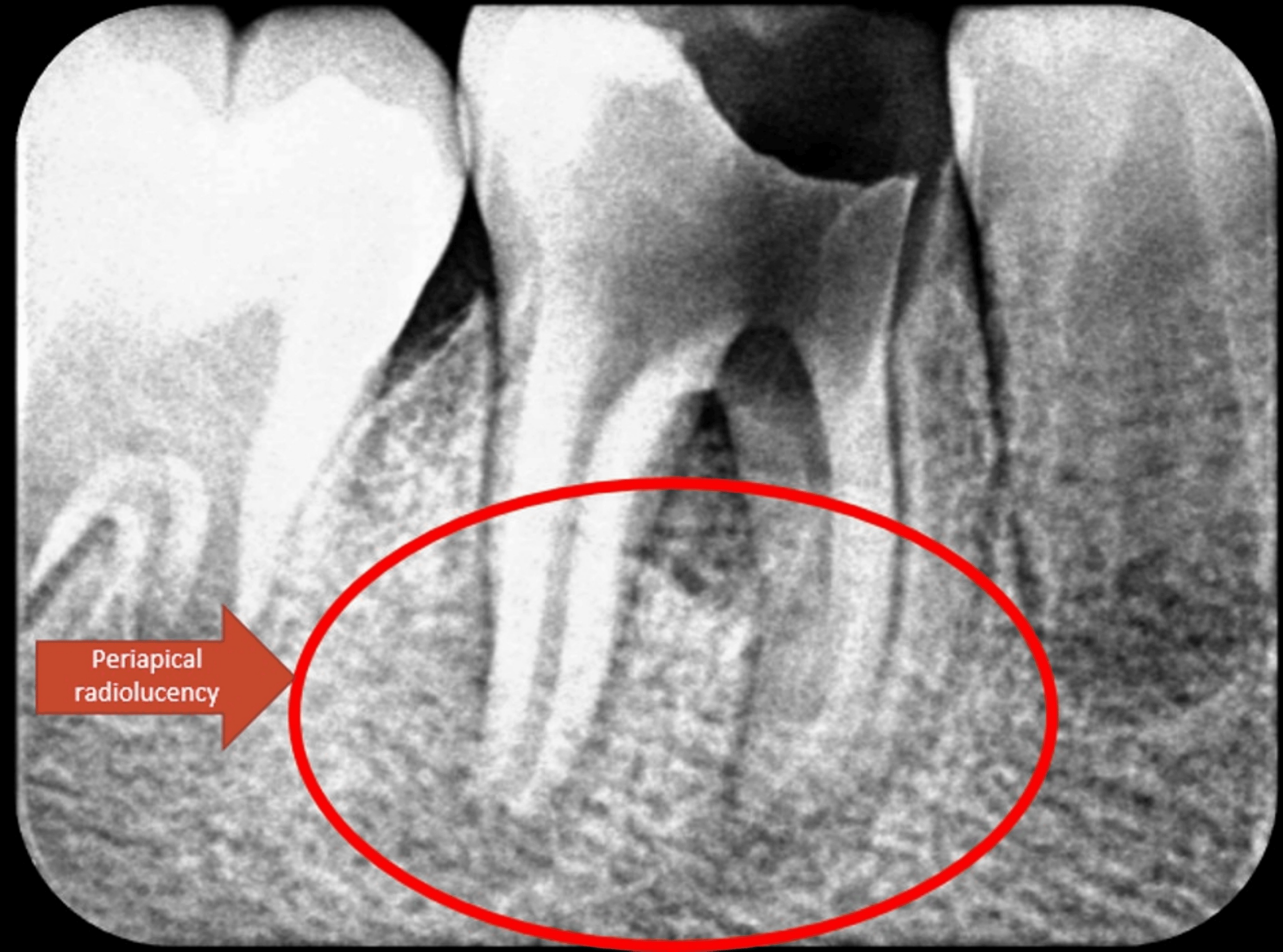
Pre-treatment intra-oral periapical (IOPA) radiograph with respect to 46 showing occlusal radiolucency involving enamel, dentine and pulp, periodontal ligament widening, furcation involvement with inter-radicular and periapical radiolucency

A 10-year-old boy presented to the outpatient department with a grossly decayed tooth in the lower right posterior region of the jaw. Both medical and dental histories were non-contributory. Extraoral examination revealed submandibular lymphadenopathy. Intraoral examination showed that tooth 46 had deep occluso-proximal caries involving the enamel, dentin, and pulp (Figure 1). The intraoral periapical radiograph showed an occlusal radiolucency due to caries involving the enamel, dentin, and pulp, along with periodontal ligament (PDL) widening, furcation involvement, and inter-radicular and periapical radiolucency. The apical foramen appeared open in all canals (Figure 2).

After thorough evaluation of radiographic and clinical situation, the diagnosis of 46 was asymptomatic chronic irreversible pulpitis with apical periodontitis, and the prognosis of tooth deemed to be poor due to aforementioned clinical and radiographic findings (Table 1). After discussion with parents, who were reluctant to consider extraction, an attempt to restore the compromised tooth was chosen with an endodontic intervention. It was decided to use triple antibiotic paste (TAP) as an intracanal medicament, followed by obturation and repairing the perforation with biocompatible material (Biodentine™). Caries Risk Assessment for Treatment (CRAFT) assessment was done for the patient and based on the score, dietary recommendations were given to the patient.

**Table 1:** Findings with respect to 46 PDL: periodontal ligament

Tooth number	Visual examination	Tenderness to soft tissue palpation	Tenderness on percussion	Mobility	Radiographic examination	Diagnosis
46	Cavitated occluso-proximal caries	Absent	Vertical percussion-present	Absent	Occlusal radiolucency involving enamel, dentin and pulp, PDL widening, furcation involvement with inter-radicular and peri-apical radiolucency	Asymptomatic chronic irreversible pulpitis with apical periodontitis

**Figure 3:**
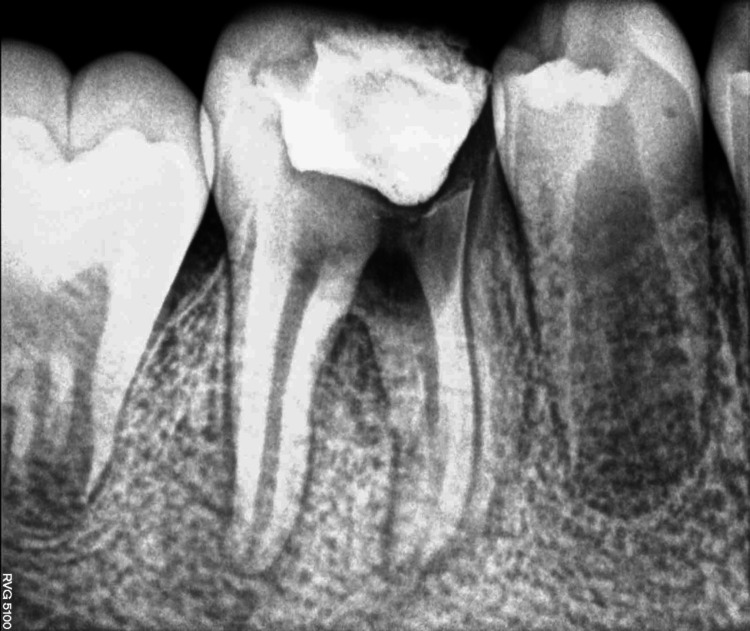
Pathological perforation in the pulpal floor near the furcation area with respect to 46

**Figure 4:**
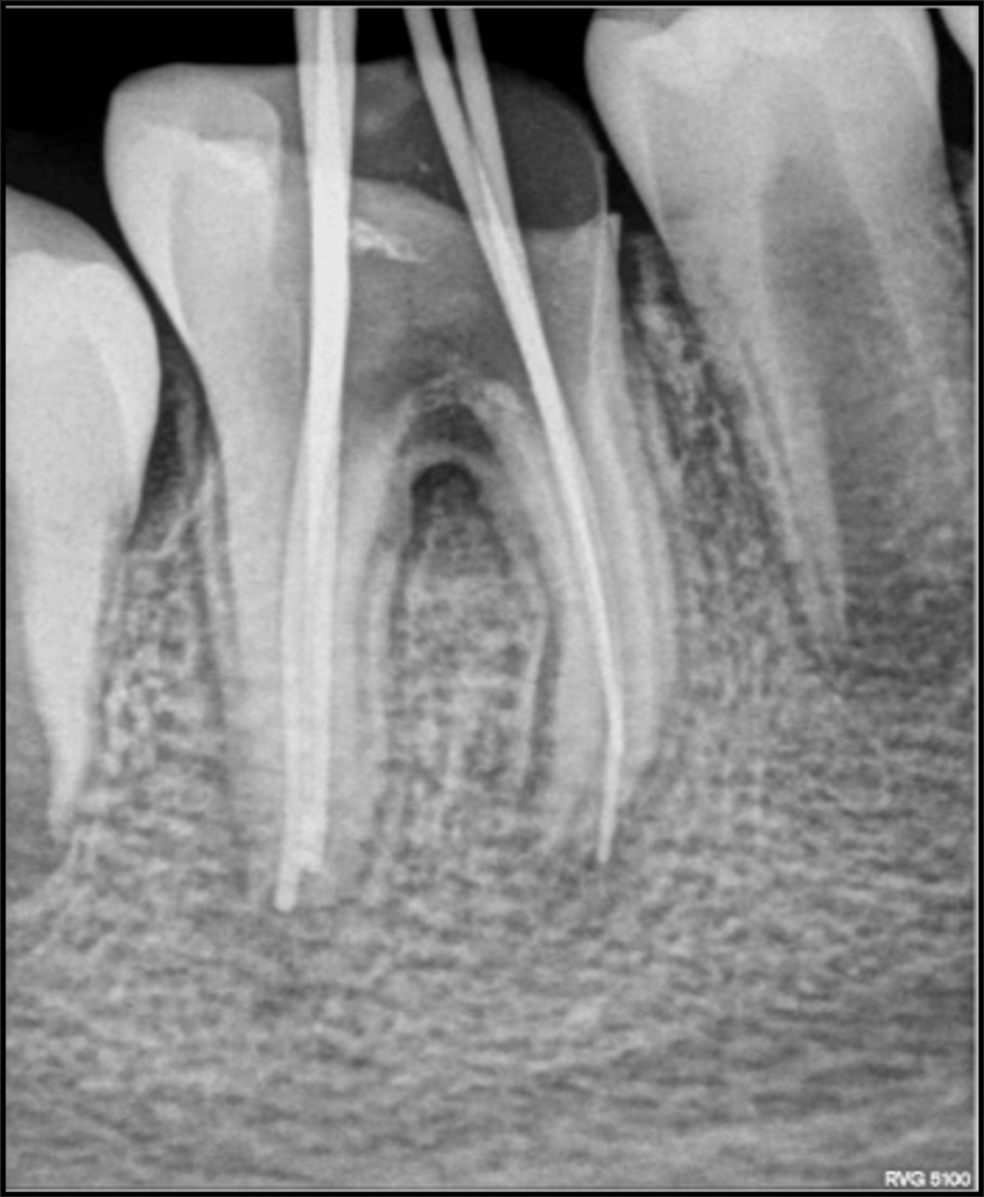
Intraoral periapical (IOPA) radiograph to check mastercone with respect to 46

After obtaining written parental consent, local anesthesia (2% lignocaine with 1:80,000 adrenaline) was administered using the inferior alveolar nerve block (IANB) technique. Access cavity preparation was done with respect to 46 using a large round bur (BR 31). During the procedure, a perforation was detected in the pulpal floor near the furcation area, which was later confirmed through radiographic evaluation (Figure 3). The working length was determined using the conventional method without an apex locator, and biomechanical preparation was carried out using a hand filing system with standard hand K-files (No. 15 to No. 35) in sequential order. Intraoral periapical radiograph (IOPA) was taken to check the mastercone fit (Figure 4).

Preparation of triple antibiotic paste (TAP)

A critical aspect of the LSTR technique is the formulation and application of triple antibiotic paste. The most widely used combination, originally proposed by Hoshino [9], consists of Metronidazole, Ciprofloxacin, and Minocycline. In this case, we utilized commercially available tablets: Ciprofloxacin 500 mg, Metronidazole 400 mg, and Minocycline 100 mg.

The enteric coatings of tablets were removed aseptically and then pulverized into powder form using a sterile mortar and pestle. The ratio, which was used to form paste, was 1:1:1, so an equal amount of crushed tablet powders by volume was dispensed using a sterile plastic dispenser. Metronidazole I.P. liquid 500 mg/100 ml was used as the vehicle. The paste was prepared to form a creamy consistency by mixing powder and metronidazole I.P. as a vehicle. The prepared mixture was carried into the canals with the help of reamers and the tooth was temporized with zinc oxide eugenol (ZOE).

**Figure 5:**
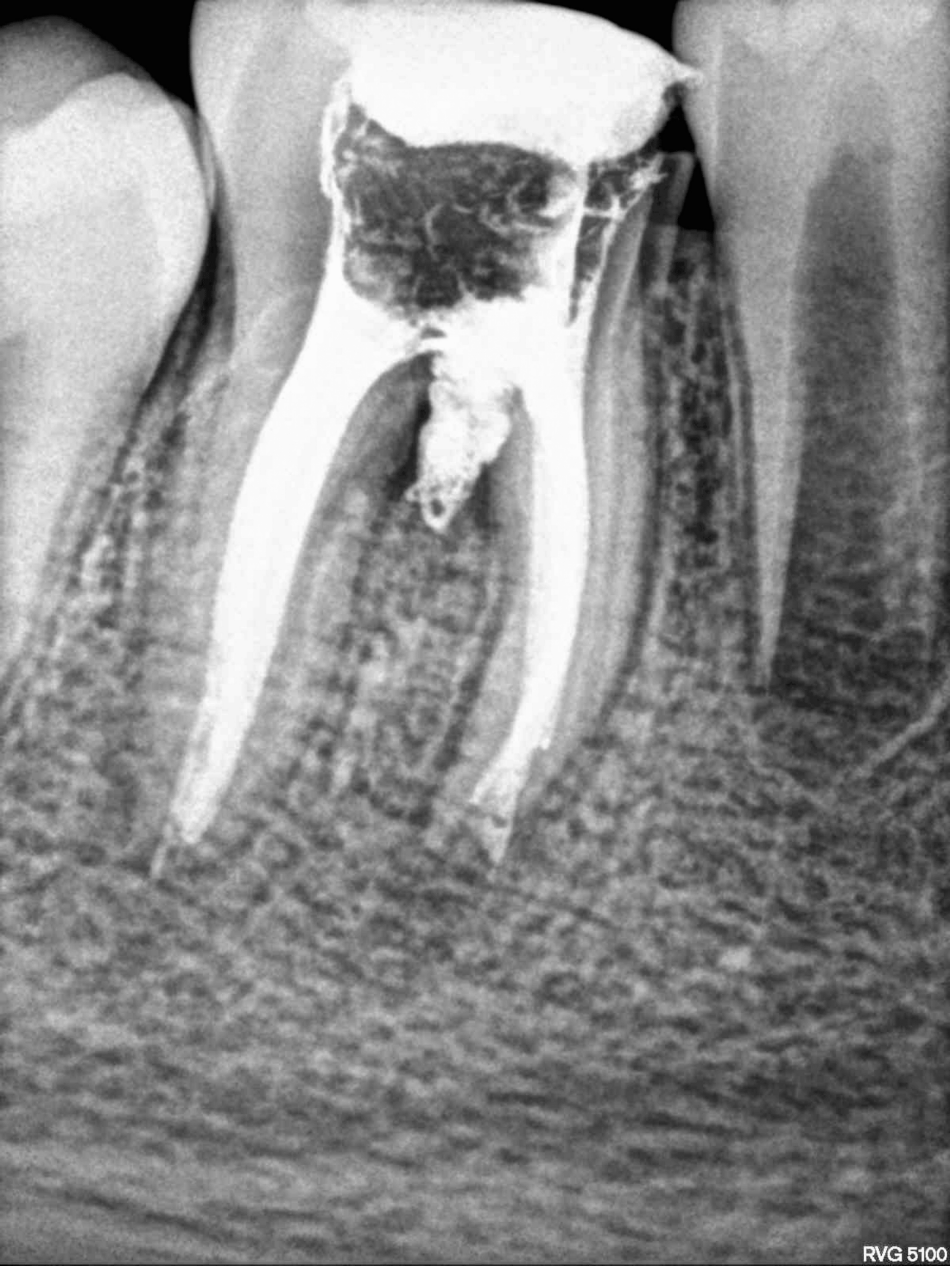
Post-obturation intraoral periapical (IOPA) radiograph with respect to 46

The follow-up was done to assess healing at one week and four weeks for clinical condition and flare-up evaluation. Radiographic evaluation at four weeks revealed adequate healing of the periapical tissues and evidence of bone formation. Therefore, the obturation was done using Gutta-percha points (GP points) and Endoflas FS™ (Sanlor Laboratories, Miami, FL, USA) as an endodontic sealer (Figure 5).

**Figure 6:**
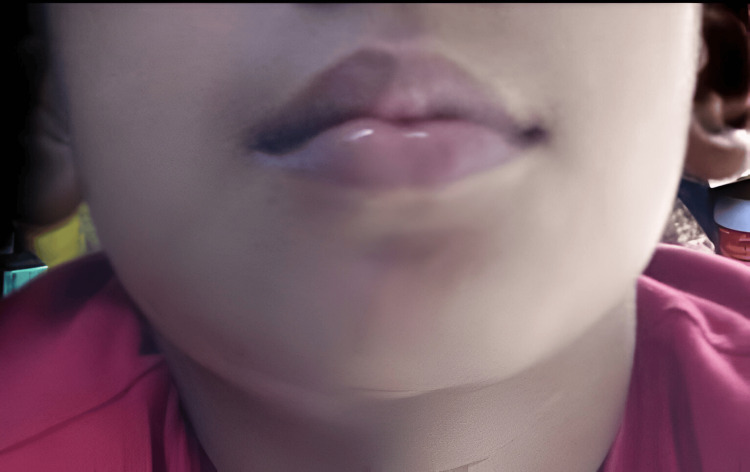
Extraoral photograph showing swelling in the lower right back region of jaw with respect to 46 three days post-obturation

Three days post-obturation, the patient reported with asymptomatic extra-oral swelling in the lower right back region of the jaw (Figure 6). On extra-oral examination, a diffuse swelling was seen on the right middle and lower third of the face. Palpation revealed a non-tender, non-compressible and non-fluctuant swelling, pain on percussion was negative and mobility was absent. Radiographic assessment revealed extrusion of an endodontic sealer material through the perforation site in the furcation area. The patient was prescribed an oral antibiotic (Amoxycillin + Clavulanic acid 457 mg BID for five days and Metrogyl 200 TID for five days) and oral analgesic for pain management (Ibuprofen 200 mg SOS).

**Figure 7:**
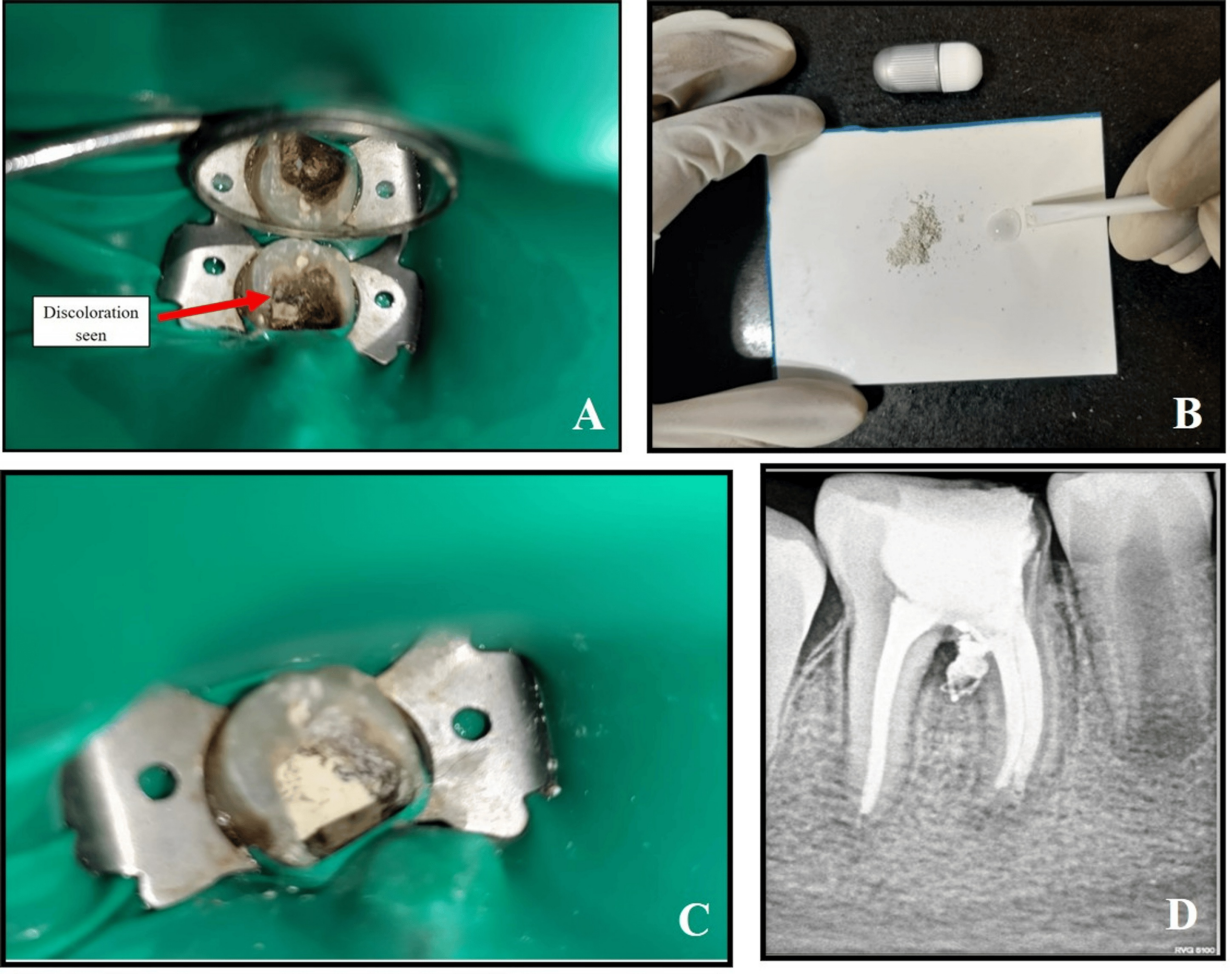
Repair of perforation site with Biodentine (Septodont™) with respect to 46 A: Pre-treatment clinical image under complete rubber dam isolation B: Mixing of Biodentine™ following the instructions C: Placement of Biodentine™ at the perforation site for repair under complete rubber dam isolation D: Intra-oral periapical (IOPA) radiograph immediate post-treatment

The swelling resolved uneventfully within three days, after which the perforation site was sealed using Biodentine™, and the tooth was temporarily restored with glass ionomer cement (GIC) (Figure 7). Two weeks later, a definitive coronal restoration was completed using a preformed stainless-steel crown (SSC) (3M ESPE, St. Paul, MN, USA).

The following table (Table 2) provides a visit-wise summary of the treatments carried out using the materials listed (Table 3).

**Table 2:** Summary of visit-wise treatment done TAP: Triple antibiotic paste; SSC: Stainless-steel crown

Visits	Treatment performed
1^st^ visit	OPD, Oral prophylaxis
2^nd^ visit	46-Access opening done. Working length determination, biomechanical preparation, preparation and insertion of TAP in canals.
3^rd^ visit	1-week recall and check-up
4^th^ visit	4-week recall and check-up
5^th^ visit	Obturation done
6^th^ visit	3 days later, the patient reported with an extra-oral swelling.
7^th^ visit (1 week later)	Furcal repair with Biodentine.
8^th^ visit	2 weeks later for definitive restoration using SSC
9^th^ visit	1 week follow-up post SSC
10^th^ visit	6-month follow-up
11^th^ visit	1-year follow-up
12^th^ visit	2-year follow-up

**Table 3:** List of materials used in the treatment

Sr no.	Materials, armamentarium	Brand name	Manufactured by	Batch no., Date of Manufacture and expiry
1	Tab. Ciprofloxacin 500 mg	Ciprofloxacin hydrochloride tablet IP 500 mg	Cipla	Batch no.: AFB20H41 Mfg. date: Nov, 2020 Exp. date: Oct, 2023
2	Tab. Metronidazole 400 mg	Tab Metrogyl 400	J B Chemicals and Pharmaceuticals Ltd	Batch no.: DM21049 Mfg. date: April, 2021 Exp. date: March, 2025
3	Tab. Minocycline 100 mg	Minocycline Hydrochloride Capsule Ip 100 mg	Omega Pharma	Batch no.: PC2146 Mfg. date: Feb, 2021 Exp. date: Jan, 2023
4	Metronidazole I.P	Flagyl IV	ASHP	Batch no.: AZ1430 Mfg. date: Feb, 2021 Exp. date: March, 2023
5	Biodentine	Biodentine^TM^	Septodont	Batch no.: G6243 Mfg. date: Oct, 2020 Exp. date: Nov, 2023
6	Endoflas	Endoflas F S	Sanlor laboratories	
7	Stainless steel crown	Crown stainless steel Permanent molar	3M ESPE	
8	Mortar and pestle			

Follow-up

**Figure 8:**
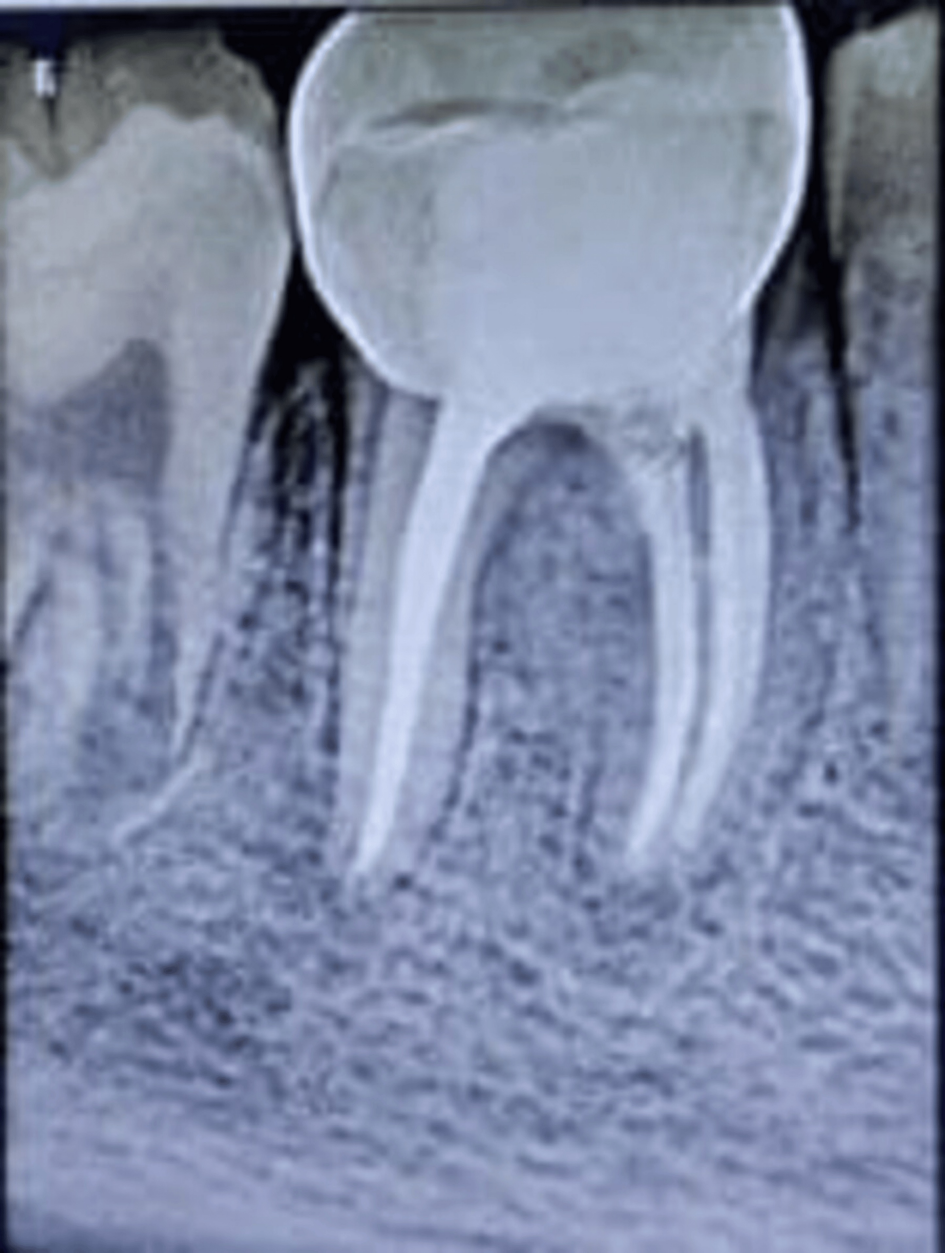
Two-year follow-up radiograph with respect to 46

One-week follow-up after SSC placement with respect to 46 revealed no signs of inflammation, pain, or sensitivity clinically. At the six-month follow-up, the patient remained asymptomatic. At the two-year follow-up, radiographic evaluation showed complete resorption of the extruded sealer along with evidence of bone formation (Figure 8).

## Discussion

Modern medicine emphasizes disease prevention through tissue preservation, reversal, and regeneration [12], with surgical intervention considered only when conservative management fails [13]. The success of non-surgical endodontic treatment relies on proper cleaning, disinfection using intracanal medicaments, and effective obturation of the root canal system [14].

Systemic antibiotic efficacy depends on patient compliance and is limited by the first-pass effect, which reduces drug bioavailability before reaching target tissues [15]. In periapical infections with compromised blood supply, especially in necrotic teeth, systemic delivery may be ineffective. Thus, topical antibiotic application within the root canal may offer a more effective antimicrobial approach. Different materials have been used as intracanal medicament for disinfection of infected root dentin. Calcium hydroxide, as an intracanal medicament, has been used since its introduction by Herman in 1920 due to its antibacterial properties, but the fact that it induces necrosis of healthy tissues and promotes dystrophic calcification cannot be ignored, so the regeneration/repair of healthy tissues is questionable [16-18].

In the present case, tooth 46 was diagnosed with asymptomatic irreversible pulpitis with apical periodontitis and a guarded to poor prognosis due to bone loss, furcation involvement, and PDL widening. Treatment options including endodontic therapy, guided tissue regeneration, and potential future extraction were discussed with the parents, who opted for endodontic management. Triple antibiotic paste (TAP), as proposed by Hoshino, was used as an intracanal medicament [19]. Its mechanism involves inhibition of collagenases and matrix metalloproteinases (MMPs), elevation of anti-inflammatory cytokines (e.g., IL-10), and stimulation of fibroblast activity by metronidazole, ciprofloxacin, and minocycline [20, 21].

The clinical and radiographic assessment of the tooth was observed at weekly recall. At a four-week interval, the normal trabecular bone pattern was observed. The patient remained clinically asymptomatic, with no reported pain or swelling. Radiographic evaluation showed signs of healing, including interradicular bone formation with a dense trabecular pattern, a decrease in periodontal ligament (PDL) widening, and inhibition of root resorption. This was indicative of the success of triple antibiotic paste therapy, which was similar to the case report published by Deolikar et al. [22]. The time interval between the occurrence of the perforation and its repair should not exceed six months [23]. Therefore, we opted to perform obturation first, followed by perforation repair using tricalcium silicate cement, as described by Asgary [24]. However, this approach was contrary to the recommendations [25, 26].

Obturation was performed using Endoflas FS™, a zinc oxide eugenol-based material with calcium hydroxide and iodoform, known for its antimicrobial properties, hydrophilicity, and suitability in pediatric cases due to its resorbability [27]. Following obturation, the patient experienced an endodontic flare-up attributed to the extrusion of sealer through the perforation site, which further compromised the prognosis without any neurological symptoms. Unlike those reported by Dalopoulou et al. [28], this paper emphasizes the use of triple antibiotic paste (TAP), a 1:1:1 combination of ciprofloxacin, metronidazole, and minocycline as an effective intracanal disinfectant in the management of compromised first permanent molars. TAP provides broad-spectrum antimicrobial activity, particularly in polymicrobial infections, and has demonstrated superior efficacy compared to calcium hydroxide, especially against resistant bacteria such as Enterococcus faecalis. Supported by studies like Hoshino and endorsed by the American Association of Endodontists (AAE), TAP also promotes tissue repair and regeneration. In the present case, TAP was selected over conventional medicaments due to the complexity of the infection and furcation involvement, contributing to favorable clinical and radiographic outcomes. Literature supports its efficacy in infection control and tissue repair, as demonstrated by Vijayaraghavan et al. [3], Utneja et al. [21], Reynolds et al. [29], and Deolikar et al. [22].

One of the concerns with the use of antibiotics is the potential for bacterial resistance, especially when administered systemically in high doses. Although the volume of medicaments used in this therapy was minimal and no adverse effects were reported, caution is advised in patients with known sensitivities to the components of triple antibiotic paste (TAP), including its active pharmaceutical ingredients or excipients. The few limitations with the use of triple antibiotic paste are discoloration, which is mainly caused by minocycline [29]. The decolorization by the Tetracycline family is due to a photo-initiated reaction. It binds with calcium ions via chelation to form insoluble complexes. So, the usage should be limited to the root canal only. Discoloration in the pulp chamber was very significant in the present case report, which was a challenge for obturation. So that’s why in the present report, perforation repair was carried out later because of difficulty in visualizing the canal orifices, and also due to the chances of orifices getting blocked with the perforation site repair material. Additionally, emerging biomaterials have demonstrated promise in enhancing the repair of iatrogenic perforations in primary molars, suggesting their potential as preferred options in pediatric dental care [30].

Here in the present case report, a definitive coronal seal was obtained with preformed stainless steel crowns as an interim restoration which may be replaced later once permanent occlusion stabilizes, to replace it with a suitable metallic or aesthetic extra-coronal restoration (such as a porcelain fused to metal crown) depending on the effect of TAP on staining, choice of the patient in terms of aesthetics and other considerations (successful outcome at stage of repeat prosthesis, crown height, gingival health, costs effectiveness, etc.).

The successful outcome in the present case depends on case selection with informed consent, protocol of disinfection by TAP and irrigation, repair of furcal perforation with biomimetic material, follow-up, and most importantly, the patient’s willingness in terms of both compliance and repair.

## Conclusions

The success of endodontic treatment relies heavily on the complete eradication of bacteria from the root canal system. Re-infection can occur if microorganisms persist in the periapical tissues or if the coronal seal is compromised. Based on the current literature and the clinical outcomes demonstrated in this case, it can be concluded that in young permanent first molars affected by extensive caries, furcation involvement, periodontal challenges, root resorption, and mobility, a conservative treatment approach utilizing triple antibiotic paste (TAP) for canal disinfection and Biodentine™ for furcation repair offers a viable alternative to extraction. This approach provides a favorable prognosis while minimizing the risk of periodontal and orthodontic complications associated with premature tooth loss.

## Figures and Tables

**Figure 9:**
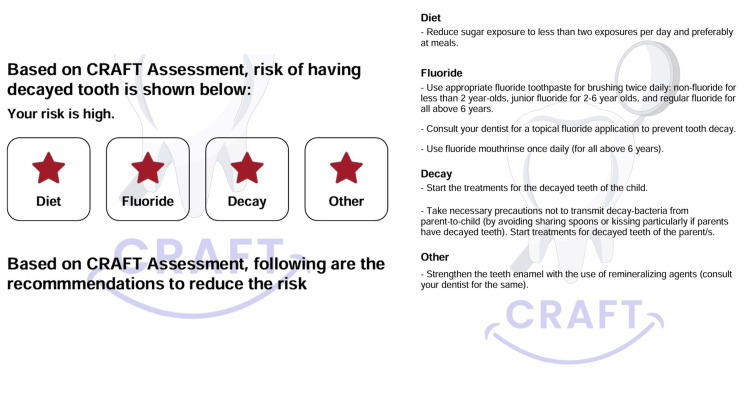
Caries Risk Assessment for Treatment (CRAFT) a digital android app tool used for caries risk assessment and dietary recommendations for the patient based on the CRAFT score Image credit: CRAFT tool (Digital android app) conceptualized by Dr. Ashwin Jawdekar. Copyright © LITTLE SMILES CHILDCARE PVT. LTD., used with permission.
